# Vertical transmission of the entomopathogenic soil fungus *Scopulariopsis brevicaulis* as a contaminant of eggs in the winter tick, *Dermacentor albipictus*, collected from calf moose (New Hampshire, USA)

**DOI:** 10.1080/21501203.2019.1600062

**Published:** 2019-04-05

**Authors:** Jay A. Yoder, Benjamin M. Rodell, Lucas A. Klever, Cameron J. Dobrotka, Peter J. Pekins

**Affiliations:** a Department of Biology, Wittenberg University, Springfield, OH, USA;; b Department of Natural Resources and the Environment, University of New Hampshire, Durham, NH, USA

**Keywords:** Internal mycoflora, endosymbiont, elk tick, horse tick, *Alces alces*, United States

## Abstract

Moose naturally acquire soil fungi on their fur that are entomopathogenic to the winter tick, *Dermacentor albipictus*. Presumed to provide a measure of on-host tick control, it is unknown whether these soil fungi impact subsequent off-host stages of the tick. Eggs and resultant larvae originating from engorged, adult female winter ticks collected from dead calf moose (*Alces alces*) were used to investigate the presence and extent of fungal infection. Approximately 40% of eggs and larvae were infected, almost exclusively by the fungus *Scopulariopsis brevicaulis* (teleomorph *Microascus brevicaulis*: Microascaceae, Ascomycota). Eggs analysed on the day of oviposition and day of hatching had high frequency (40%) of *S. brevicaulis*, whereas the frequency in eggs harvested *in utero* was minimal (7%); therefore, exposure occurs pre-oviposition in the female’s genital chamber, not by transovarial transmission. At hatching, larvae emerge containing *S. brevicaulis* indicating transstadial transmission. Artificial infection by topical application of eggs and larvae with a large inoculum of *S. brevicaulis* spores caused rapid dehydration, marked mortality; pathogenicity was confirmed by Koch’s postulates. The high hatching success (>90%) and multi-month survival of larvae imply that *S. brevicaulis* is maintained as a natural pathobiont in winter ticks.

## Introduction

The utilisation of a single moose (*Alces alces*) by the winter tick *Dermacentor albipictus*, a one-host species, exposes winter ticks to numerous on-host fungi that are acquired by moose from soil in breeding wallows and bed sites. This exposure is presumed adaptive as a natural, bio-control of on-host tick abundance (Yoder et al. 2018a, 2018b). These fungi are common soil saprobes and anamorphs of ascomycetes, that also act as tick pathogens (entomopathogens) by producing copious amounts of spores as the primary transmission route of infection (Fernandes et al. 2012). Exposure to and subsequent infection by these fungi play an important ecological role in tick abundance and distribution. In the mycotic cycle, fungal spores affix to the cuticular surface of ticks and germinate. The resulting hyphae gain internal access by penetrating the mouth, anus, spiracles, glandular openings, and soft tissues between leg segments. The fungus then establishes within, fixing on the tick as an ecological niche; typically, only a single fungus is recovered internally (Cradock and Needham 2011; Yoder et al. 2017). Massive dehydration is a classic sign of fungal infection in ticks and is characterised by: (1) an increase in measurable whole body water loss rate, (2) retraction and curling of the legs, particularly in a box-like pattern, (3) uncoordinated movements, (4) inability to self-right, and (5) deflation of the eggshell chorion and arrested development as identified by lack of visible internal guanine (primary excretory waste material in ticks; Sonenshine 1991) in the developing embryo. Upon death, the hyphae have enveloped the tick within a mycelium that creates a mouldy “mummy”.

*Alternaria, Aspergillus* (mainly *A. flavus), Cladosporium* (mainly *C. cladosporioides), Fusarium, Mucor, Paecilomyces, Penicillium*, and *Scopulariopsis* have been recovered internally from on-host stages of *D. albipictus* (New Hampshire and Maine, USA populations) collected from moose skin and fur (Yoder et al. 2017, 2018b). These fungi have been isolated from fed larvae (as pharate nymphs; i.e. “molters”), non-fed and fed nymphs, and non-fed and fed females as evidence of fungal infection of winter ticks on host moose. Eggs and hatched larvae, off-host life stages found on the ground, have not been examined rigorously for the presence of internal fungal infection.

Eggs are laid after the large, blood-engorged adult female drops off the moose to the ground. After a brief crawling period, the adult female settles in a moisture-rich shelter to lay eggs that hatch about a month post-oviposition. Unfed larvae emerge at the ground surface and are quiescent for the remainder of summer. In early autumn, larvae are triggered by short-day cues (Yoder et al. 2016) and subsequently climb vegetation and quest for a host moose. It is important to examine eggs and unfed larvae for fungi, in that natural entomopathogenic fungal infection could influence off-host survival and viability of eggs and unfed larvae; the latter, the only stage that infests moose (Samuel 2004; Jones et al. 2017, 2018; Ellingwood 2018).

The purpose of this study was to determine the internal mycoflora of eggs and unfed larvae, with the goals of: (1) examining the extent that winter tick eggs and unfed larvae are infected with fungi as a residual of the adult female tick, (2) identifying fungi that infect winter tick eggs and larvae, specifically whether a single, select fungus or mixture of fungal components are disease-causing, and (3) assessing the capacity for artificial infection by a topically applied large spore inoculum that serves to confirm pathogenicity to the egg and larval stages. Overall, we tested the hypothesis that exposure to on-host fungi through the adult female tick subsequently may impact the off-host egg and larval stages.

## Materials and methods

### Eggs and unfed larvae, experimental materials

Experimental materials and chemicals were purchased from Fisher Scientific (Pittsburgh, PA) unless otherwise noted. All culture work was performed with Potato Dextrose Agar (PDA) + 0.05% chloramphenicol in 100 × 15 mm Petri plates and was performed in a laminar flow hood (Cole-Palmer, Vernon Hills, IL). Sterilisation was by autoclave (121ºC, 19 psi, 15 min), Bunsen burner flame, 95% ethanol, or purchased sterile from the manufacturer. Powder-free gloves were worn while handling ticks, and soft forceps (Featherweight narrow tip forceps, DR Instruments, Bridgeview, IL) were used as needed. *Metarhizium anisopliae* strain F52 was isolated from Met52® (Novozymes Biologicals, Salem, VA) and served as a control.

Eggs and their resultant unfed larvae originated from wild, engorged adult female ticks collected (within 24 h of death) from carcasses of three calf moose in Milan, NH, April, 2017 (co-author P. Pekins holds collection permits). These engorged females were collected by hand from inside both hind legs of the calves. They were loosely attached indicating completion or near completion of feeding, and were removed with slightest touch. Each (total *n* = 62) was placed into a mesh-covered 50 mL polypropylene centrifuge tube and stored at 93% RH (Wharton 1985), 25 ± 0.5ºC, and light (L): dark (D) cycle of 8 h:16 h for oviposition. Eggs were then transferred to a clean centrifuge tube prior to hatching. Samples of > 20 larval specimens were selected at random, mounted on slides, and identified as *Dermacentor albipictus* using keys (Lindquist et al. 2016).

### Fungus isolation and identification

Specimens were surface sterilised by three, 1 min rinses, each in a mild bleach solution of DI (deionised) water:ethanol:5.25% NaOCl (18:1:1 v/v/v), followed by two fresh DI water rinses (Yoder et al. 2008). Each specimen was sectioned with a scalpel, the body portions were covered with cooled molten PDA, and the agar was allowed to solidify. The agar plates were incubated in darkness at 25 ± 0.5ºC. Body portions were examined daily with 40/45× light microscopy to identify growing hyphae. Subcultures of hyphal tips (1 hypal tip/plate PDA) were made from those hyphae originating internally from within the body; each hyphal tip was considered an isolate. The identical set of protocols was performed to establish a control.

Fungi were identified ~ 2 weeks after isolation (1000× microscopy under oil) after spore and phialide characteristics appeared on subcultures grown on PDA, Czapek Dox agar, and cornmeal agar (Samson et al. 1988; Barnett and Hunter 2003). Confirmation of identification (microscopic and/or molecular) was carried out at University of Alberta Microfungus Collection and Herbarium, University of Toronto, Toronto, CAN, and University of Cincinnati Microfungus Collection, Department of Biological Sciences, University of Cincinnati, Cincinnati, OH. Fungal isolates are deposited at University of Cincinnati, Cincinnati, OH (specimen lot UC112018) and Wittenberg University, Springfield, OH (specimen lot WU-6-XI2018).

The number of specimens that tested positive for fungi were expressed as the mean percent ± SE, based on 10 replicates of 10 specimens each (total *n* = 100, randomly selected from adult female ticks/moose calves). Data were compared using an analysis of covariance (ANCOVA; *p* = 0.05) following a log-it transformation (JMP, SAS Institute, Cary, NC).

Three age-cohort groups of eggs were analysed: (1) eggs obtained by dissection *in utero* from adult females (8 days pre-oviposition) that were surface-sterilised and rinsed with DI water, (2) eggs obtained on the day of oviposition when females oviposited directly into the mild bleach surface sterilising solution (a fed female was positioned over a 6 dram glass vial, eggs were subsequently rinsed in DI water), and (3) eggs that had been surface sterilised and DI water rinsed 14 days post-oviposition (mid-point to hatching). Three age-cohorts of unfed larvae (surface sterilised and rinsed with DI water) were also analysed: (1) newly emerged, teneral larvae (on the day of hatching), (2) larvae at 14 days post-hatching (cuticle fully sclerotized), and (3) larvae at 5 months post-hatching (i.e. typical questing age).

From the same batch of adult females, one set of eggs and larvae was used for fungal isolation, and a second set to track development. These were placed individually into a 1.5 mL mesh-covered micro-centrifuge tube and stored at 93% RH and 25ºC. Dead larvae were identified by failure to respond to prodding, lack of leg movement, legs typically curled into a box-like pattern, and a deflated opisthosoma. Dead eggs were identified by a deflated and collapsed eggshell chorion, and a non-developing embryo was identified by lack of visibly increasing accumulation of internal guanine.

### Confirmation of fungus infection

Pathogenicity was determined by inducing an artificial infection, measuring water loss rate, and from re-isolation of the treatment fungus from cadavers; all are consistent with the “dehydration hypothesis” associated with mycoses and Koch’s postulates (Cradock and Needham 2011; Willis et al. 2011; Yoder et al. 2017). Experimental eggs were round, full, and showed signs of guanine accumulation, and larvae could self-right and crawl five body lengths; both were 14 days of age in the experiment.

Artificial infection was induced by topical treatment with an aqueous fungal inoculum. The inoculum concentration was 1.3 × 10^7^ spores/mL made in phosphate buffered saline (PBS, pH 7.5) + 0.05% Tween 20 (Fisher) from spores scraped from 2-week-old cultures (Greengarten et al. 2011; Yoder et al. 2017). Spore count of the inoculum was adjusted using an AO Spencer Bright-Line hemocytometer (St. Louis, MO) and 0.1% trypan blue exclusion. The culture was chosen at random for preparation of an inoculum: three different cultures from a specific fungus were used to ensure that the same fungus was not used in all experimental replicates. Fungi isolated from eggs were used to treat eggs, and fungi isolated from larvae were used to treat larvae; the control was PBS + 0.05% Tween.

Ten specimens were treated simultaneously in 1 mL of inoculum in a 1.5 mL micro-centrifuge tube by gentle agitation for 1 min. After treatment, each specimen was placed individually into a clean, mesh-covered 1.5 mL tube and stored at 80% RH (Wharton 1985), 25°C, in a 8 h:16 h L:D cycle within a glass desiccator. Specimens were examined under 40–45× light microscopy after 10 days. Dead specimens were subsequently surface sterilised, dissected, and the body contents were examined for presence and identification of fungi as described above. A total of 100 eggs and 100 larvae were examined in the treatment and control groups (*n* = 10 replicates of 10 specimens each). Data were expressed as the mean ± SE. Mortality data were compared with ANCOVA (*p* = 0.05; Abbott correction, log-it transformation), and survival time with the Student *t*-test (*p* = 0.05).

To determine water loss rate, specimens were weighed individually with a micro-balance (SD ± 0.2 μg precision, ± 6 μg accuracy at 1 mg; Cahn Ventron Co., Cerritos, CA). Specimens were treated with the inoculum as described above and stored at 93% RH, 25°C, at 8 h:16 h L:D cycle. Four days after the fungal treatment, each specimen was weighed and transferred to a glass desiccator at 0% RH (calcium sulphate; W. A. Hammond Drierite Co., Xenia, OH); the rate of water loss is exponential at this condition (Wharton 1985). Each specimen was weighed singly five times to determine mass. After the final measurement, each was dried to completion at 90°C (<± 2°C; Blue M Electric Co., Chicago, IL). The percent body water content was calculated as: 100% (*f* – *d*)/*f*, where *f* is the initial (fresh) mass, *d* is the dry mass. Each original mass measurement was converted to a water mass (*m*) value by subtracting the dry mass. The water loss rate (integumental + respiratory water loss) was calculated as: *m_t_* = *m*_0_exp-*kt*, where *m_t_* is the water mass at any time *t, m*_0_ is the initial water mass, and -*kt* is the water loss rate (Wharton 1985). The same specimens were used to determine percent body water content and rate of water loss rate. Water content was compared using ANCOVA (*p* 0.05; log-it transformation). Sample size for each treatment and control group was 100 (*n* = 10 replicates of 10 specimens each). Water loss rates in experimental groups were compared by testing for similarity of slopes among several regressions (ANCOVA; *p* = 0.05).

## Results

### Fungus isolation and identification

The number of fungus-infected eggs was lowest *in utero* (7%) compared to at oviposition (52%) and hatching (38%) (*p* < 0.05 in each pairwise comparison; Table 1). *Scopulariopsis brevicaulis* was isolated frequently (71–97%), mostly occurring as the sole isolate in specimens (Table 1); it was in an anamorphic state at each stage. *Aspergillus* and *Penicillium* were also recovered (3–14%), co-occurring with *S. brevicaulis in utero* and at hatching (Table 1). A 98% aseptic efficiency was achieved when the culturing manipulations (*n* = 100) were replicated without a specimen. Specimens tracked from the same cohort in the fungal analysis developed on schedule with 94.4 ± 1.2% eggs hatching; resultant larvae kept at 93% RH and 25°C experienced ≤ 10% mortality after 6 months. Fungal infection of larvae was similar at hatching, at 14 days, and at 5 months (40–46%, Table 2). As with eggs, *S. brevicaulis* was the predominant fungus representing 95–100% of isolated fungi at each age; *Aspergillus* and *Penicillium* were also isolated (2–5%, Table 2).

**Table 1.** Internal mycoflora of eggs of *Dermacentor albipictus*. -, not determined or not detected. Culture control, identical manipulation for plating a specimen except without using a specimen. Data (the mean ± SE) followed by the same superscript letter within a column do not differ significantly from each other. *n* = 10 replicates of 10 eggs each.

	Developmental stages of egg:			
Fungus	*In utero*	At oviposition	14 days after oviposition	Culture control
%/100 eggs containing fungi	7.2 ± 2.5^a^	52.1 ± 5.7^a^	38.1 ± 6.1^a^	
% fungi identified:				
*Aspergillus* spp.		5.8 ± 2.0^b^	-	-
*Penicillium* spp.	14.3 ± 1.1^b^	-	2.6 ± 1.5^b^	-
*P. expansum*	14.3 ± 1.1^b^	-	-	-
*Scopulariopsis brevicaulis*	71.4 ± 2.7^c^	94.2 ± 1.4^c^	97.4 ± 1.6^c^	-
*Trichoderma* spp.				2/100 (100%)

**Table 2.** Internal mycoflora of unfed larvae of *Dermacentor albipictus*. -, no isolates. Data (the mean ± SE) followed by the same superscript letter within a column do not differ significantly from each other. *n* = 10 replicates of 10 larvae each.

	Developmental stages of larva:		
Fungus	At hatching	14 days after hatching	5 months after hatching
%/100 larvae containing fungi	45.9 ± 7.1^a^	42.7 ± 5.6^a^	39.7 ± 5.2^a^
% fungi identified:			
*Aspergillus flavus*	-	4.7 ± 0.2^b^	-
*Penicillium* spp.	2.2 ± 0.1^b^	-	-
*Scopulariopsis brevicaulis*	97.8 ± 1.2^c^	95.3 ± 1.7^c^	100.0^b^

The experimental data presented in Tables 1 and 2 originated from winter ticks collected from one moose (calf 1); interestingly, alike data from the other two calves varied somewhat. The proportions of infected 14-day-old eggs (44.2 ± 3.4%) and 5-month-old larvae (41.6 ± 3.6%) originating from ticks collected from calf 3 were similar to those from calf 1 (*p* > 0.05). Ticks from calf 2 yielded a lower proportion of infection of 14-day old eggs (24.3 ± 4.3%) and 5-month old larvae (31.7 ± 5.5%) (*p* < 0.05). As with calf 1, the predominant fungus in both eggs and larvae from calves 2 and 3 was *S. brevicaulis* (95.2 ± 4.1% combined), with *Aspergillus, A. flavus, Cladosporium cladosporioides*, and *Penicillium* isolated occasionally. Approximately 92.7 ± 2.2% of eggs and larvae that originated from calves 2 and 3 (pooled) developed on schedule, hatched successfully, and the resultant larvae survived > 6 months with low mortality (≤7%).

### Confirmation of fungus infection

Mortality was higher in eggs and larvae treated topically with 10^7^ *S. brevicaulis* spore/mL inoculum compared to the saline-treated controls (*p* < 0.05 in each pairwise comparison; Tables 3 and 4). In all cases, mortality in the *M. anisopliae* group was significantly higher (*p* < 0.05) than in the *S. brevicaulis* group. Carcasses of specimens treated with *S. brevicaulis* had high internal recovery of *S. brevicaulis*; similar results occurred in the *M. anisopliae* group (Tables 3 and 4). Mortality was higher in larvae than eggs in the *S. brevicaulis* treatment; a similar effect occurred in the *M. anisopliae* group (*p* < 0.05 for each fungus).

**Table 3.** Effect 10 days post-treatment with *Scopulariopsis brevicaulis* aqueous inoculum on healthy eggs of *Dermacentor albipictus*. PBS control, pH 0.5 phosphate buffered saline + 0.05% Tween. Data (the mean ± SE) followed by the same superscript letter within a column do not differ significantly from each other. *n* = 10 replicates of 10 eggs each.

Experimental group	Egg stages for treatment:	
	At oviposition	14 days after oviposition
%/100 eggs that failed to hatch		
PBS control	11.3 ± 2.4^a^	7.9 ± 1.3^a^
*S. brevicaulis* [1.3 × 10^7^ spores/mL]	54.2 ± 3.0^b^	62.0 ± 4.1^b^
*M. anisopliae* [1.3 × 10^7^ spores/mL]	71.5 ± 2.8^c^	67.4 ± 2.4^c^
% dead eggs positive for a fungus		
PBS control	36.4 ± 1.9^d^	25.0 ± 1.1^d^
*S. brevicaulis* [1.3 × 10^7^ spores/mL]	72.2 ± 2.6^c^	66.1 ± 2.3^c^
*M. anisopliae* [1.3 × 10^7^ spores/mL]	75.0 ± 3.7^c^	68.7 ± 3.1^c^

**Table 4.** Effect 10 days post-treatment with *Scopulariopsis brevicaulis* aqueous inoculum on healthy unfed larvae of *Dermacentor albipictus*. PBS control, pH 0.5 phosphate buffered saline + 0.05% Tween. Data (the mean ± SE) followed by the same superscript letter within a column do not differ significantly from each other. *n* = 10 replicates of 10 specimens each.

Experimental group	Larval stages for treatment:		
	At hatching	14 days after hatching	5 months after hatching
%/100 dead larvae			
PBS control	24.1 ± 2.1^a^	15.8 ± 3.4^a^	19.1 ± 2.8^a^
*S. brevicaulis* [1.3 × 10^7^ spores/mL]	72.8 ± 4.2^b^	67.8 ± 1.9^b^	66.1 ± 2.5^b^
*M. anisopliae* [1.3 × 10^7^ spores/mL]	83.1 ± 2.3^c^	86.8 ± 3.4^c^	81.1 ± 3.1^c^
% Dead larvae positive for a fungus			
PBS control	33.3 ± 2.0^d^	37.5 ± 2.2^d^	42.1 ± 3.1^d^
*S. brevicaulis* [1.3 × 10^7^ spores/mL]	82.2 ± 2.6^c^	77.9 ± 2.3^c^	83.3 ± 2.6^c^
*M. anisopliae* [1.3 × 10^7^ spores/mL]	91.6 ± 2.7^e^	94.3 ± 3.1^e^	88.9 ± 2.3^c^

Eggs and larvae treated with the *S. brevicaulis* spore inoculum lost proportionally more body water than saline-treated specimens (*p* < 0.05), but less than specimens treated with *M. anisopliae* (Tables 5 and 6, *p* < 0.05). Overall, water loss rate was higher in larvae than eggs (*p* < 0.05).

**Table 5.** Effect of *Scopulariopsis brevicaulis* aqueous inoculum [1.3 × 10^7^ spores/ml] on water balance characteristics of healthy eggs of *Dermacentor albipictus*. PBS control, pH 0.5 phosphate buffered saline + 0.05% Tween. Data (the mean ± SE) followed by the same superscript letter within a column do not differ significantly from each other. *n* = 10 replicates of 10 specimens each.

Egg stages for treatment	Water balance characteristic of egg 4 days post-application:			
	Fresh mass (mg)	Water mass (mg)	Water content (%)	Water loss rate (%/h)
At oviposition				
PBS control	0.074 ± 0.006^a^	0.051 ± 0.004^a^	68.92 ± 0.71^a^	0.70 ± 0.02^a^
+ *S. brevicaulis*	0.060 ± 0.008^a^	0.040 ± 0.003^a^	66.67 ± 0.51^a^	1.04 ± 0.03^b^
+ *M. anisopliae*	0.069 ± 0.011^a^	0.045 ± 0.005^a^	65.23 ± 0.64^a^	1.31 ± 0.03^c^
14 days after oviposition				
PBS control	0.064 ± 0.012^a^	0.040 ± 0.002^a^	62.50 ± 0.44^b^	0.87 ± 0.021^d^
+ *S. brevicaulis*	0.067 ± 0.014^a^	0.041 ± 0.005^a^	61.19 ± 0.61^b^	1.36 ± 0.030^c^
+ *M. anisopliae*	0.070 ± 0.009^a^	0.046 ± 0.005^a^	65.71 ± 0.49^a^	1.61 ± 0.043^e^

**Table 6.** Effect of *Scopulariopsis brevicaulis* aqueous inoculum [1.3 × 10^7^ spores/ml] on water balance characteristics of healthy unfed larvae of *Dermacentor albipictus*. PBS control, pH 0.5 phosphate buffered saline + 0.05% Tween. Data (the mean ± SE) followed by the same superscript letter within a column do not differ significantly from each other. *n* = 10 replicates of 10 specimens each.

Larval stages for treatment	Water balance characteristic of larva 4 days post-application:			
	Fresh mass (mg)	Water mass (mg)	Water content (%)	Water loss rate (%/h)
At hatching				
PBS control	0.058 ± 0.007^a^	0.040 ± 0.003^a^	68.97 ± 1.34^a^	1.20 ± 0.05^a^
+ *S. brevicaulis*	0.061 ± 0.006^a^	0.041 ± 0.003^a^	67.21 ± 1.24^a^	1.69 ± 0.03^b^
+ *M. anisopliae*	0.054 ± 0.012^a^	0.035 ± 0.005^a^	64.81 ± 1.16^a^	2.91 ± 0.03^c^
14 days after hatching				
PBS control	0.039 ± 0.008^b^	0.023 ± 0.004^b^	58.97 ± 1.21^b^	1.72 ± 0.04^b^
+ *S. brevicaulis*	0.043 ± 0.012^b^	0.027 ± 0.006^b^	62.79 ± 1.09^b^	2.82 ± 0.04^c^
+ *M. anisopliae*	0.042 ± 0.005^b^	0.026 ± 0.005^b^	61.90 ± 1.10^b^	4.91 ± 0.03^d^
5 months after hatching				
PBS control	0.041 ± 0.009^b^	0.023 ± 0.007^b^	56.10 ± 1.05^b^	1.61 ± 0.03^b^
+ *S. brevicaulis*	0.044 ± 0.006^b^	0.026 ± 0.005^b^	59.09 ± 1.24^b^	3.54 ± 0.05^e^
+ *M. anisopliae*	0.040 ± 0.010^b^	0.023 ± 0.004^b^	57.50 ± 1.30^b^	4.46 ± 0.04^d^

Egg mass (fresh), body water content (and water mass to dry mass, *m*/*d*) declined relative to increasing water loss as age increased (*p* < 0.05 in each pairwise comparison); larvae also had age-related differences in water balance characteristics (*p* < 0.05). There were no significant differences in percent body water content, fresh mass, water mass, and *m*/*d* within a particular age-related cohort of eggs and larvae. In all cases, dry mass correlated positively with water mass (*R* ≥ 0.91; *p* < 0.001). Thus, the differences in water loss rate within an age cohort were due to the *S. brevicaulis* treatment and not differences in surface area:volume ratio.

## Discussion

Our study indicates that the mitosporic fungus *S. brevicaulis* is “inherited” in the wild from adult female (mothers) *D. albipictus*. Under aseptic, sterile conditions of oviposition and hatching, eggs and resultant larvae containing *S. brevicaulis* were identified via internal fungal cultures. *S. brevicaulis* exists within the egg and larva in almost a pure culture in its anamorphic state, whereas *Aspergillus, Cladosporium*, and *Penicillium*, highly antagonistic fungal genera, were found only on rare occasion. Despite ~ 40% prevalence of *S. brevicaulis* in eggs and resultant larvae, nearly all infected eggs hatched, and larvae survived for multiple months in the laboratory when stored at 93% RH and 25°C. A similar outcome (i.e. internal *S. brevicaulis*, lack of other internal fungi, complete survival) was found with the lone star tick *Amblyomma americanum*, American dog tick *Dermacentor variabilis*, and brown dog tick *Rhipicephalus sanguineus* (Benoit et al. 2005; Yoder et al. 2008), but not the blacklegged tick *Ixodes scapularis* (Boston, MA specimens, Benoit et al. 2005; Grand Island, NY specimens, A. P. LeBarge, 2018, Wittenberg University, Springfield, OH, unpublished observations). Similar evidence of maternal transmission of *S. brevicaulis* was previously reported for *D. variabilis* (Benoit and Yoder 2004) and *R. microplus* (Camargo et al. 2012), and we extend this conclusion to include the winter tick *D. albipictus*. These studies together indicate that, although egg and larval infection by *S. brevicaulis* is common in multiple tick species, it likely persists as non-harmful in natural populations.

The most likely source of *S. brevicaulis* in eggs and larvae is from exposure of adult winter ticks to contaminated moose fur and skin. Animals acquire this fungus from exposure to soil where *S. brevicaulis* functions as a saprobe (Sharma and Choudhary 2014; Verekar and Deshmukh 2017; Zhang et al. 2017). *S. brevicaulis* is a frequent coloniser of animal hair and skin (non-dermatophyte) due to its keratinophilic and keratinolytic nature. Prevalence in our samples was ~20–50% in eggs and larvae, mid-range of previous measurements of ~80% in *D. variabilis* (Yoder et al. 2008) and ~20% in *R. sanguineus* (Benoit and Yoder 2004). In all cases, eggs and larvae from *S. brevicaulis*-positive populations developed normally with long-term, multi-month survival in the laboratory.

In this study, only the adult females (mothers) had direct contact with moose fur and skin, whereas, eggs and larvae harbouring *S. brevicaulis* were raised in sterile micro-centrifuge tubes. As expected, the relative concentration of *S. brevicaulis* in forest soils varies locally, even within moose range in the adjacent states of New Hampshire and Maine in the north-eastern United States (Yoder et al. 2018a); correspondingly, the amount of *S. brevicaulis* varies on the fur and skin of sex-age cohorts of moose (Yoder et al. 2018b). We conclude that the infection rate of *S. brevicaulis* in eggs and larvae of *D. albipictus* will vary depending on the individual host moose and its relative exposure to the soil fungus.

The fact that few eggs harvested *in utero* tested positive for *S. brevicaulis* suggests that exposure and acquiring *S. brevicaulis* by trans-ovarial transmission is unlikely. The typical qualifying threshold signifying trans-ovarial transmission is > 70% infected eggs as early embryos (modified from Macaluso et al. 2002). We report < 10% of *D. albipictus* eggs *in utero* contained *S. brevicaulis*. The proportion of *S. brevicaulis*-positive eggs increases substantially by the day of oviposition, suggesting that eggs become contaminated within the mother’s genital chamber. This contamination possibly occurs from spores of *S. brevicaulis* residing in the cuticular glands associated with the genital chamber, direct penetration by hyphae through the ovipore, or the male introducing *S. brevicaulis* spores during mating (Benoit and Yoder 2004). Because internal culturing indicates that the proportion of *S. brevicaulis* is similar in eggs and larvae, the proportion of contaminated eggs presumably yields a similar proportion of contaminated larvae through trans-stadial transmission.

There is little doubt that *S. brevicaulis* is potentially disease-causing to eggs and larvae of *D. albipictus* if transmission occurs by way of a large inoculum of spores (our treatment) that produces an artificial, lethal infection (Yoder et al. 2017). Although the entomopathogenic potential of *S. brevicaulis* is documented (Suleiman et al. 2013), *S. brevicaulis* is not recommended in biocontrol of ticks because it can cause opportunistic skin infections in people (Fernandes et al. 2012). In contrast to natural populations where pathogenic effects to ticks are not observed, the *S. brevicaulis* inoculum treatment caused mortality identified by high rates of desiccation associated with abnormal water loss rate, mouldy cadavers, and re-isolation of *S. brevicaulis* from dead specimens per Koch’s postulates. Treatment with an inoculum of lower spore concentration of *S. brevicaulis* has no debilitating or lethal effect on *D. variabilis* or *R. microplus* (Yoder et al. 2008; Camargo et al. 2012). Indeed, our and other studies found no apparent, damaging effect on ticks in nature from harbouring *S. brevicaulis* in specific study populations.

Further, internal occurrence of *S. brevicaulis* in ticks may actually be beneficial by providing resistance against secondary fungal infections. For example, laboratory studies indicate that ticks harbouring *S. brevicaulis* receive protection when challenged against low inoculums of the entomopathogenic fungus *M. anisopliae* (Yoder et al. 2008; Camargo et al. 2012). Such a natural resistance mechanism might possibly enhance egg viability and off-host larval survival of *D. albipictus* that ultimately influence field abundance of ticks and infestation levels on moose (Jones et al. 2017, 2018; Ellingwood 2018). Because this fungus is capable of causing tick mycosis artificially in the laboratory, but under field conditions likely causes minimal harm and might benefit ticks (symbiotic: endocommensal), *S. brevicaulis* meets the definition of a pathobiont in natural populations of winter ticks and host moose.
